# Association Between Baseline Therapy and Flare Reduction in Mepolizumab-Treated Patients With Hypereosinophilic Syndrome

**DOI:** 10.3389/fimmu.2022.840974

**Published:** 2022-04-13

**Authors:** Andreas Reiter, Guillaume Lefevre, Maria C. Cid, Namhee Kwon, Eleni Mavropolou, Steven W. Yancey, Jonathan Steinfeld

**Affiliations:** ^1^ Department of Hematology and Oncology, University Hospital Mannheim, Heidelberg University, Mannheim, Germany; ^2^ Université de Lille, Centre Hospitalier Universitaire de Lille (CHU Lille), Institut d’Immunologie, Centre de Référence National des Syndromes Hyperéosinophiliques (CEREO), Institute for Translational Research in Inflammation Infinite-U1286. Inserm, Lille, France; ^3^ Department of Autoimmune Diseases, Institut d’Investigacions Biomèdiques August Pi I Sunyer (IDIBAPS), Hospital Clínic, University of Barcelona, Barcelona, Spain; ^4^ Respiratory Research and Development, GlaxoSmithKline (GSK), Brentford, United Kingdom; ^5^ Clinical Statistics, GlaxoSmithKline (GSK), Brentford, United Kingdom; ^6^ Respiratory Therapeutic Area, GlaxoSmithKline (GSK), Research Triangle Park, NC, United States; ^7^ Respiratory Research and Development, GlaxoSmithKline (GSK), Collegeville, PA, United States

**Keywords:** hypereosinophilic syndrome, eosinophils, clinical immunology, mepolizumab, oral corticosteroids, immunosuppressive therapy, biologics

## Abstract

**Background:**

Current standard-of-care treatments for hypereosinophilic syndrome (HES) include oral corticosteroids (OCS) and immunosuppressive/cytotoxic (IS/CT) therapies. The anti-IL-5 monoclonal antibody mepolizumab has also recently been approved for patients with this disease. The objective of this analysis was to assess the relationship between baseline therapy and flare reduction in patients with HES treated with mepolizumab, using data from the Phase III 200622 study (NCT02836496).

**Methods:**

In the double-blind, parallel-group 200622 study, eligible patients were ≥12 years old and had HES for ≥6 months, ≥2 flares in the previous 12 months, blood eosinophils ≥1000 cells/μL at screening and ≥4 weeks’ stable HES therapy. Patients were randomised (1:1) to receive mepolizumab 300 mg subcutaneously or placebo every 4 weeks for 32 weeks plus their existing HES therapy. This *post hoc*, descriptive analysis assessed the effect of baseline HES therapy [IS/CT (± OCS), OCS No IS/CT, and No IS/CT/OCS] on the proportion of patients with ≥1 flare during the study period, the annualised rate of flares, time to first flare, and the proportion of patients with ≥1 flare during Weeks 20─32, with mepolizumab versus placebo.

**Results:**

Mepolizumab treatment was associated with a decrease in the proportion of patients who experienced ≥1 flare during the study period in all baseline therapy groups versus placebo (32–96% reduction). Similarly, the probability of a flare was lower with mepolizumab (14.3–31.4%) than placebo (35.7–74.1%) in all baseline therapy groups, as was the annualised flare rate (0.22–0.68 vs 1.14–1.62). The proportion of patients who experienced ≥1 flare during Weeks 20–32 was reduced with mepolizumab versus placebo for all baseline therapy groups (55–85% reduction). For all endpoints, the greatest effect of mepolizumab treatment was seen in the IS/CT (± OCS) group.

**Conclusions:**

Patients with poorly controlled HES are likely to achieve clinical benefit with mepolizumab in terms of flare reduction, regardless of their baseline therapy.

**Clinical Trial Registration:**

(https://clinicaltrials.gov/ct2/show/NCT02836496).

## Introduction

Hypereosinophilic syndrome (HES) is a rare and debilitating multisystem disorder characterised by elevated eosinophil counts in the peripheral blood and/or tissues and eosinophil-mediated organ damage, without a secondary cause for the eosinophilia (1). Disease presentation is heterogeneous; any tissue or organ system can be affected, although skin, lung, and gastrointestinal involvement are the most common manifestations (2). The disease course is also heterogeneous, with different patterns of relapse and disease activity, and patients may experience flares, a worsening of HES-related disease activity requiring an increase in treatment (3). As disease outcomes in HES are linked to the nature and extent of end-organ damage (4, 5), treatment goals focus on the prevention and reversal of organ damage to improve symptoms by reducing eosinophilic inflammation (6).

Treatment options for HES vary depending on the heterogeneous underlying drivers of disease. For example, patients who are positive for the fusion gene *FIP1L1-PDGFRA* respond effectively to imatinib (tyrosine kinase inhibitor) treatment (2, 6). However, current standard of care treatment for patients with HES without the *FIP1L1-PDGFRA* fusion gene or other tyrosine kinase rearrangements is less effective and includes oral corticosteroids (OCS) as a first-line treatment often used with immunosuppressive and/or cytotoxic (IS/CT) therapies, commonly hydroxycarbamide or interferon-α (2, 6). Depending on the pattern of the disease course, treatments may be used chronically or for the treatment of flares as required (3). In many cases in which chronic treatment is indicated, OCS therapy is discontinued or used in combination therapy owing to adverse side effects or lack of efficacy. Many CT therapies and IS therapies are also associated with significant toxicity and side effects, and have variable clinical efficacy (2). Therefore additional therapeutic options for HES, including OCS sparing treatments, are needed.

Mepolizumab is a humanised monoclonal antibody that binds to and inactivates interleukin (IL)-5, thereby reducing eosinophil haematopoiesis and survival (7, 8). By doing so, mepolizumab is able to reduce blood eosinophil counts to within normal physiological limits (9). Mepolizumab is an approved add-on therapy for a number of eosinophilic diseases, including severe eosinophilic asthma, chronic rhinosinusitis with nasal polyps, eosinophilic granulomatosis with polyangiitis (EGPA) and HES in multiple regions worldwide (10, 11). The double-blind, Phase III 200622 study (NCT02836496), demonstrated that mepolizumab significantly reduced the incidence of disease flares, fatigue severity and blood eosinophil counts versus placebo in patients with HES, with a favourable safety profile (9).

It is currently not known whether the efficacy of mepolizumab treatment in HES is affected by prior use of other therapies such as OCS or IS/CT therapies. We therefore conducted a *post hoc* analysis of data from the 200622 study to assess the relationship between baseline HES therapy and mepolizumab-associated flare reduction.

## Materials and Methods

### Study Design and Patients

This was a *post hoc* analysis of data from the 200622 study (NCT02836496), a randomised, placebo-controlled, double-blind, parallel-group, multicentre, Phase III trial, full details of which have been reported previously (9). Briefly, patients were randomised (1:1) to receive mepolizumab 300 mg subcutaneously or placebo every 4 weeks for 32 weeks in addition to their existing HES therapy. Patients were ≥12 years of age at screening; had a diagnosis of HES ≥6 months before screening based on organ system involvement and/or dysfunction that could be directly related to a blood eosinophil count >1500 cells/μL on ≥2 occasions, and/or tissue eosinophilia, without a discernible secondary cause; were receiving stable HES therapy for ≥4 weeks before the baseline visit; had ≥2 flares within the past 12 months and a baseline blood eosinophil count ≥1000 cells/μL at screening. Patients maintained the same regimen of baseline treatment throughout the 32-week study unless they had a worsening of symptoms that required an increase in therapy. Patients positive for the *FIP1L1-PDGFRA* fusion gene were excluded. The trial was conducted in accordance with the ethical principles of the Declaration of Helsinki, the International Conference on Harmonization Good Clinical Practice guidelines, and applicable country-specific regulatory requirements. The local institutional review board or ethics committee at each study centre oversaw trial conduct and documentation (see ethics statement for further details). All patients provided written informed consent.

### *Post Hoc* Analysis Outcomes

The endpoints assessed in this *post hoc* analysis were the proportion of patients who experienced ≥1 flare during the 32-week study period (primary endpoint of the 200622 study) (9), the annualised rate of flares, time to first flare, and the proportion of patients with ≥1 flare during Weeks 20─32 (secondary endpoints of the 200622 study) (9). Flares were defined as: a) a HES-related clinical manifestation (based on a physician-documented change in clinical signs or symptoms) that required either an increased dose of maintenance OCS ≥10 mg prednisone equivalent/day for 5 days or an increase in/addition of any CT and/or IS HES therapy or b) receipt of ≥2 courses of blinded OCS during the treatment period. To be considered as a new flare, the onset date of a flare must have been ≥14 days after the resolution of the previous flare.

Each endpoint was analysed for patient subgroups defined by baseline HES treatment type, focusing on OCS and IS/CT use; other medications were not considered in subgroup classifications. Three treatment type subgroups were defined: IS/CT therapy both with and without OCS [IS/CT (± OCS)]; OCS without IS/CT (OCS No IS/CT); and neither OCS nor IS/CT (No IS/CT/OCS). IS/CT therapies included but were not limited to hydroxycarbamide, ciclosporin, imatinib, methotrexate, and azathioprine.

### Statistical Analysis

Patient demographic and clinical characteristics at baseline stratified by baseline HES therapy type [IS/CT (± OCS); OCS No IS/CT; No IS/CT/OCS] were analysed descriptively. The significance of differences in demographic or clinical characteristics was not tested. The proportion of patients with a flare during the 32-week study period and during Weeks 20–32 was analysed using a logistic regression analysis adjusted for baseline OCS dose. Region was not included as a covariate owing to low sample sizes in some subgroups, and patients who withdrew prematurely from the study were included in the analysis as having a flare. The rate of flares was analysed using a negative binomial generalised linear model adjusted for baseline OCS dose, treatment and observed time (as an offset variable). The probability of a flare was analysed using a Cox proportional hazards regression analysis adjusted for baseline OCS dose. The study was powered for analysis of the intent-to-treat population of the 200622 study and was not powered for *post hoc* subgroup analyses, as such significance testing was not performed. All analyses were performed using SAS software, version 9.4 (SAS Institute, Cary, NC, USA).

## Results

### Patient Population, Demographic and Clinical Characteristics

In total, 141 patients were screened for participation. Of these, 108 patients were deemed eligible and included in this analysis. HES treatment at baseline is shown in **Table 1**. In total, 21% of patients were receiving IS/CT therapies ( ± OCS), 56% were receiving OCS without IS/CT, and 23% were receiving neither. Of patients who were receiving IS/CT, the majority (17% of all patients) were also receiving OCS and 5% of patients were receiving IS/CT without OCS. The most commonly received IS/CT therapies were hydroxycarbamide (8%), methotrexate (5%), interferon alpha (5%), imatinib (5%), and ciclosporin (4%) (**Table 1**).

**Table 1:** Baseline HES treatment in the ITT population.

Baseline HES therapy, n (%)	Placebo (N = 54)	Mepolizumab 300 mg SC (N = 54)	Total (N = 108)
**Any HES therapy**	49 (91)	50 (93)	99 (92)
IS/CT* (± OCS)	9 (17)	14 (26)	23 (21)
Azathioprine	0	2 (4)	2(2)
Ciclosporin	3 (6)	1 (2)	4 (4)
Hydroxycarbamide	4 (7)	5 (9)	9 (8)
Hydroxychloroquine	0	1 (2)	1 (1)
Immunoglobulin human normal	1 (2)	0	1 (1)
Imatinib^†^	3 (6)	2 (4)	5 (5)
Interferon alpha^‡^	4 (8)	1 (2)	5 (5)
Methotrexate^§^	0	5 (9)	5 (5)
Mycophenolate mofetil	0	1 (2)	1 (1)
Tamoxifen	1 (2)	0	1 (1)
OCS No IS/CT	31 (57)	29 (54)	60 (56)
No IS/CT/OCS	14 (26)	11 (20)	25 (23)
**OCS dose, mg/day** ^║^			
0	16 (30)	14 (26)	30 (28)
0 – ≤5	11 (20)	13 (24)	24 (22)
>5 – ≤10	18 (33)	16 (30)	34 (31)
>10	9 (17)	11 (20)	20 (19)
Other HES therapy**	19 (35)	22 (41)	41 (38)

*The classification was based on the study definition; ^†^includes one patient who received imatinib mesylate; ^‡^includes one patient who received interferon-alpha-2B and two patients who received Peg-interferon alpha-2A; ^§^includes one patient who received methotrexate sodium; ^║^prednisone or equivalent dose; **other HES therapies included but were not limited to beclometasone dipropionate, formoterol fumarate, omeprazole, salbutamol, tiotropium bromide, triamcinolone acetonide and cetirizine.

CT, cytotoxic therapy; HES, hypereosinophilic syndrome; IS, immunosuppressive therapy; ITT, intent-to-treat; OCS, oral corticosteroid; SC, subcutaneous.

Details of patient demographics and clinical characteristics are shown in **Table 2**. With a mean age of 44.4 years and 44.7 years respectively, patients receiving IS/CT (± OCS) or OCS No IS/CT were younger than patients receiving No IS/CT/OCS treatment at baseline (50.7 years). The mean [standard deviation (SD)] duration of HES was shortest in the IS/CT (± OCS) group and longest for the OCS No IS/CT group [4.62 (3.253) – 5.96 (7.838) years]. The mean (SD) number of flares in the 12 months before screening was 2.3 (0.57), 3.0 (1.25), 2.4 (1.19) in the IS/CT (± OCS), OCS No IS/CT and No IS/CT/OCS groups respectively. Additionally, the No IS/CT/OCS group had the smallest proportion of patients with >2 flares. The median (range) daily OCS dose was numerically lower in the IS/CT (± OCS) group than in the OCS No IS/CT group [7.5 (0─50) and 10.0 (3─38), respectively].

**Table 2:** Baseline demographics and clinical characteristics in the ITT population by baseline treatment type.

	IS/CT (± OCS)(N = 23)	OCS No IS/CT(N = 60)	No IS/CT/OCS(N = 25)
**Age (years), mean (SD)**	44.4 (13.78)	44.7 (15.75)	50.7 (17.21)
**Female, n (%)**	12 (52)	33 (55)	12 (48)
**Duration of HES (years), mean (SD)**	4.62 (3.253)	5.96 (7.838)	5.44 (6.132)
**Number of flares in 12 months prior to screening, n (%)**			
1	1 (4)	0	0
2	13 (57)	28 (47)	21 (84)
3	9 (39)	16 (27)	1 (4)
4	0	12 (20)	1 (4)
≥5	0	4 (7)	2 (8)
Mean (SD)	2.3 (0.57)	3.0 (1.25)	2.4 (1.19)
Most bothersome HES symptom*			
Abdominal pain or bloating	6 (26)	22 (37)	12 (48)
Breathing symptoms	14 (61)	34 (57)	12 (48)
Chills or sweats	4 (17)	8 (13)	3 (12)
Muscle or joint pain	8 (35)	23 (38)	13 (52)
Nasal or sinus symptoms	10 (43)	21 (35)	10 (40)
Skin symptoms	15 (65)	26 (43)	12 (48)
**Prednisone equivalent daily dose (mg)**			
Mean (SD)	11.8 (13.86)	9.9 (6.68)	0
Median (range)	7.5 (0─50)	10.0 (3─38)	0

*Patients could report up to 3 most bothersome symptoms.

CT, cytotoxic therapy; HES, hypereosinophilic syndrome; IS, immunosuppressive; ITT, intent-to-treat; OCS, oral corticosteroid; SD, standard deviation.

There was variation between the treatment subgroups in the frequency of HES symptoms reported by patients at baseline as the three most bothersome symptoms. Among patients in the IS/CT (± OCS) and OCS No IS/CT subgroups, breathing (61% and 57%, respectively) and skin symptoms (65% and 43%, respectively) were the most commonly reported. Among patients receiving No IS/CT/OCS, muscle or joint pain (52%) was the most commonly reported symptom.

### Impact of Mepolizumab on Flare Outcomes by Baseline Treatment

**Figure 1:**
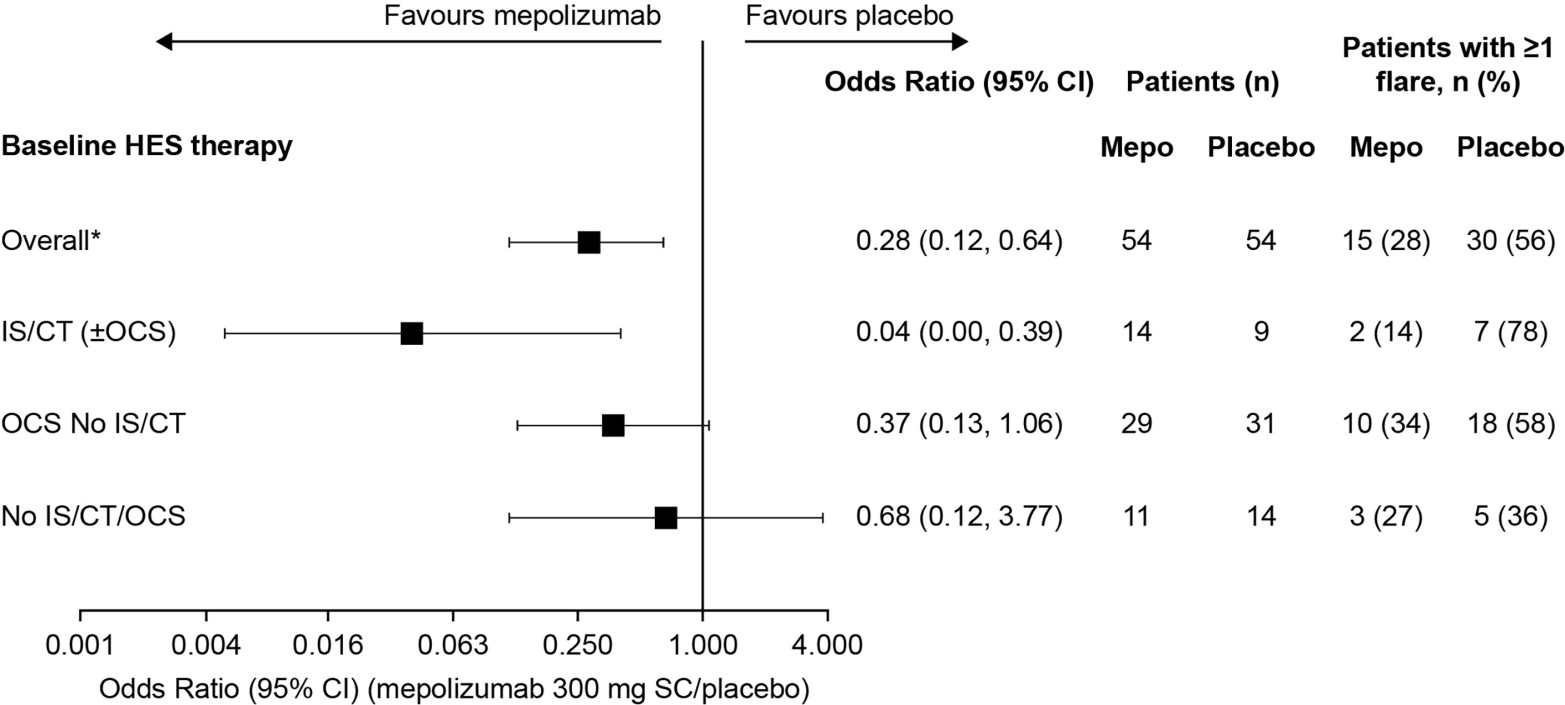
Odds of a patient experiencing ≥1 flare with mepolizumab versus placebo during the 32-week treatment period by baseline treatment type. *These values were originally reported in Roufosse et al., 2020. CI, confidence interval; CT, cytotoxic therapy; HES, hypereosinophilic syndrome; IS, immunosuppressive therapy; OCS, oral corticosteroid; SC, subcutaneous.

The proportion of patients who experienced ≥1 flare during the 32-week study period was lower among patients who received mepolizumab (range: 14–34% across the three baseline treatment groups) compared with placebo (**Figure 1**). The greatest reduction in the odds of a patient experiencing ≥1 flare with mepolizumab versus placebo was experienced in the IS/CT (± OCS) group with a 96% reduction {odds ratio [95% confidence interval (CI)]: 0.04 [0.00, 0.39]}, compared with 63% in the OCS No IS/CT group [odds ratio (95% CI): 0.37 (0.13, 1.06)], and 32% in the No IS/CT/OCS group [odds ratio (95% CI): 0.68 (0.12, 3.77)] (**Figure 1**).

**Figure 2:**
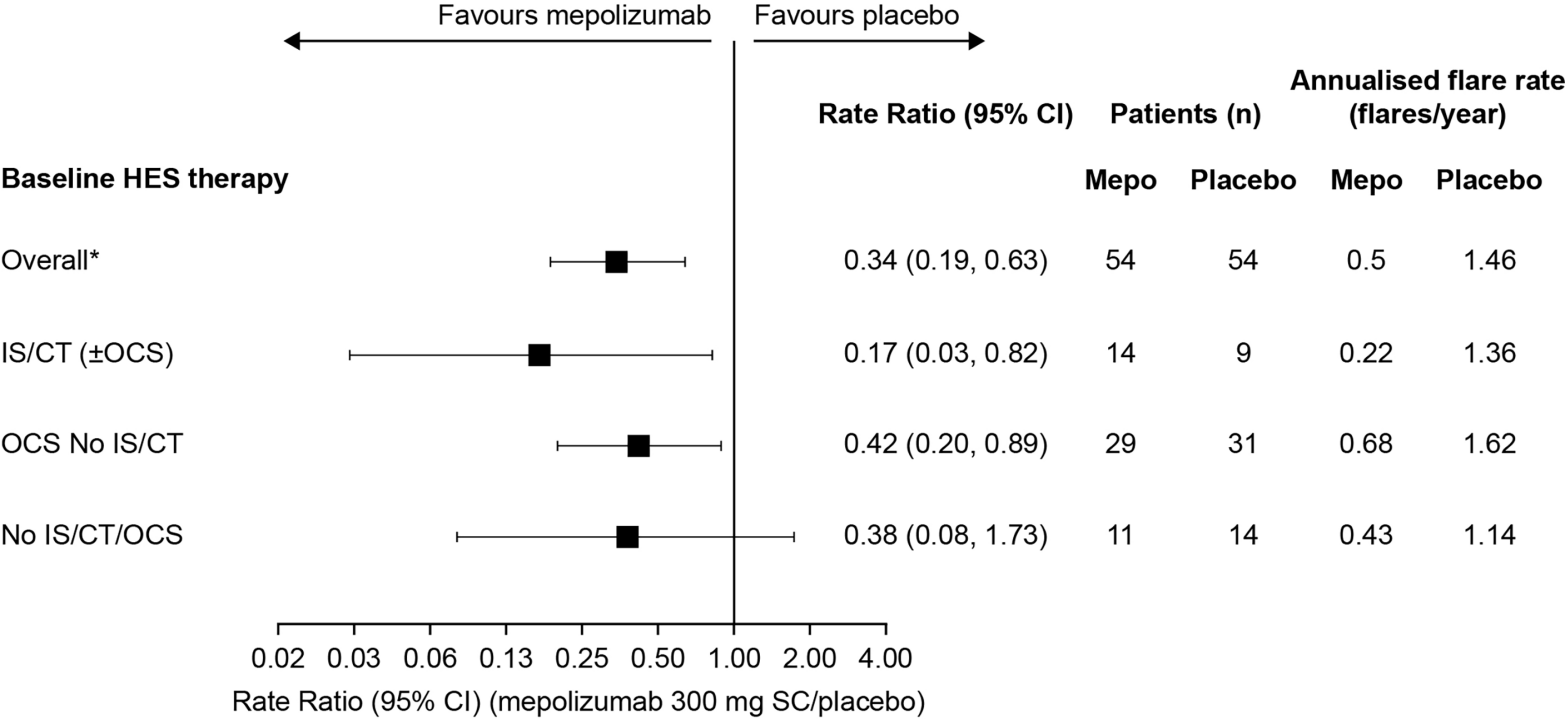
Annualised rate of flares with mepolizumab versus placebo by baseline treatment type. *These values were originally reported in Roufosse et al., 2020. CI, confidence interval; CT, cytotoxic therapy; HES, hypereosinophilic syndrome; IS, immunosuppressive therapy; OCS, oral corticosteroid; SC, subcutaneous.

The annualised rate of flares, calculated from the rate of flares during the 32-week study period was also lower with mepolizumab (0.22–0.68 flares/year across the three baseline treatment groups) versus placebo (1.14–1.62 flares/year) regardless of baseline therapy (**Figure 2**). The largest reduction was in the IS/CT (± OCS) treatment group, with an 83% reduction compared with 58% in the OCS No IS/CT group and 62% in the No IS/CT/OCS group (**Figure 2**).

**Figure 3:**
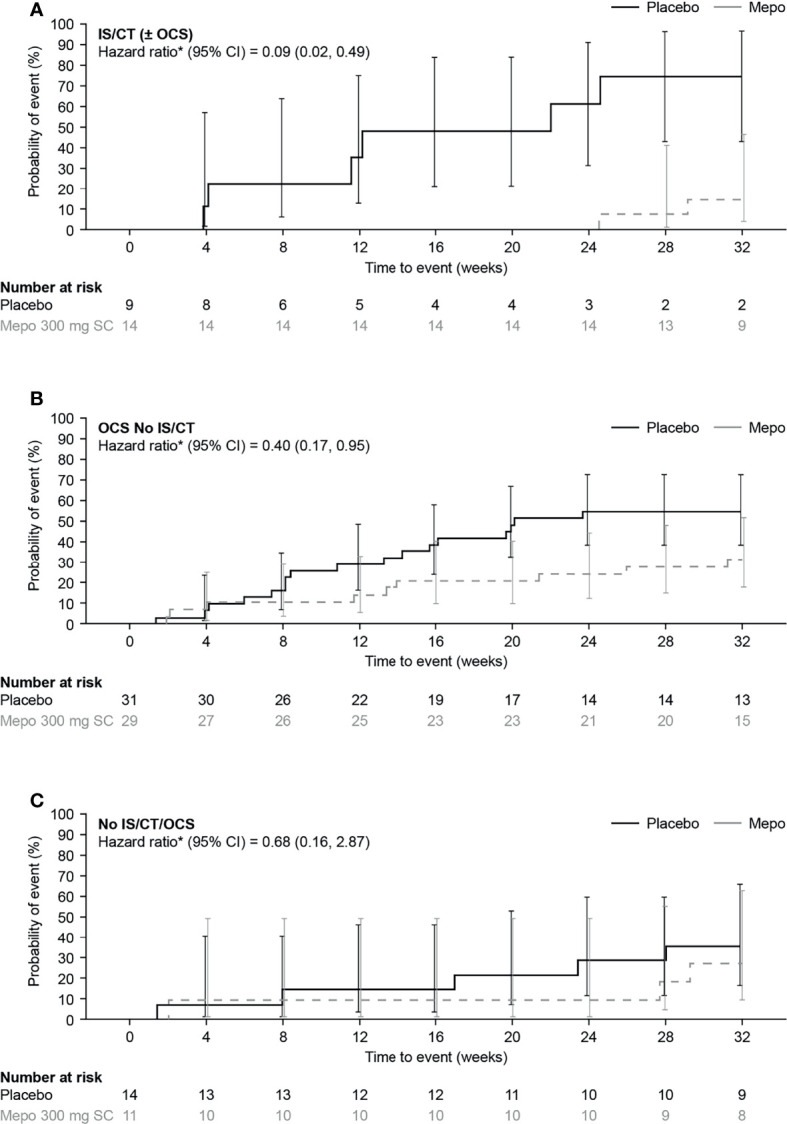
Kaplan-Meier analysis of time to first flare with mepolizumab versus placebo in **(A)** IS/CT ( ± OCS), **(B)** OCS No IS/CT and **(C)** No IS/CT/OCS subgroups. *Hazard ratio (mepolizumab 300mg SC/placebo) calculated by Cox proportional hazards regression analysis adjusted for baseline OCS dose. CI, confidence interval; CT, cytotoxic therapy; IS, immunosuppressive therapy; mepo, mepolizumab; OCS, oral corticosteroid; SC, subcutaneous.

**Figure 4:**
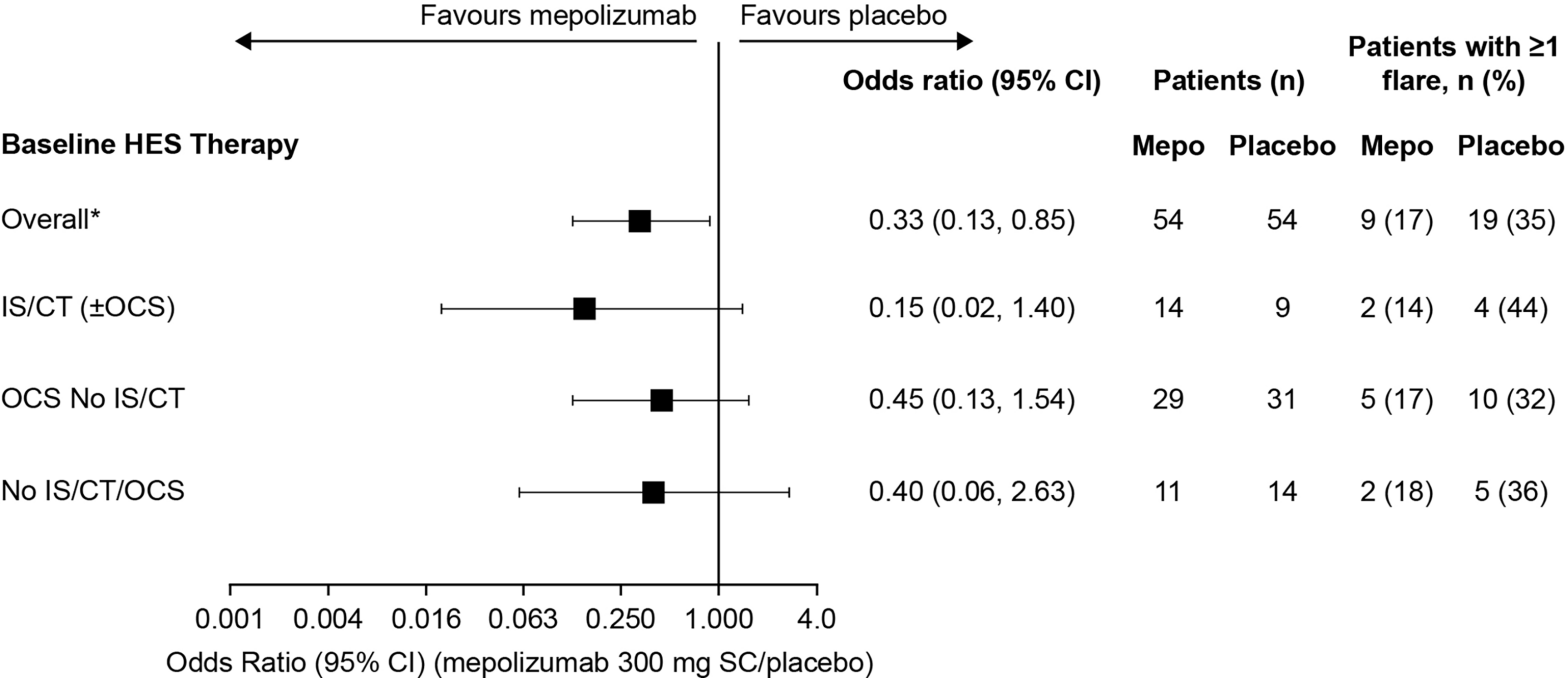
Odds of a patient experiencing ≥1 flare with mepolizumab versus placebo during Week 20 though Week 32 by baseline treatment type. *These values were originally reported in Roufosse et al., 2020. CI, confidence interval; CT, cytotoxic therapy; HES, hypereosinophilic syndrome; IS, immunosuppressive therapy; OCS, oral corticosteroid; SC, subcutaneous.

Analysis of time to first flare found that the risk of a flare by Week 32 was lower with mepolizumab versus placebo in all baseline treatment groups [91%, 60%, and 32% reduction in risk of a flare with IS/CT (± OCS), OCS No IS/CT, and No IS/CT/OCS groups, respectively] (**Figure 3**). The largest effect of mepolizumab versus placebo was seen in the IS/CT (± OCS) group where the difference between the mepolizumab and placebo groups was seen as early as Week 8 and increased with time to a 74.1% risk of a flare with placebo versus 14.3% with mepolizumab at Week 32; **Figure 3A**). In the OCS No IS/CT group the risk of a flare at Week 32 was 54.8% with placebo compared with 31.4% with mepolizumab (**Figure 3B**). The smallest difference between mepolizumab and placebo was seen in the No IS/CT/OCS subgroup (**Figure 3C**), in which the probability of flare with placebo was lower than in the other subgroups (35.7% with placebo at Week 32, vs 27.3% with mepolizumab). Finally, the proportion of patients who experienced ≥1 flare during Week 20 through Week 32 was lower with mepolizumab (14–18%) versus placebo (32–44%) in all baseline treatment groups, as evident from the lower odds of flare with mepolizumab versus placebo in the IS/CT (± OCS) group compared with the OCS No IS/CT and No IS/CT/OCS treatment groups [odds ratio: IS/CT (± OCS) = 0.15; OCS No IS/CT = 0.45; No IS/CT/OCS = 0.40; **Figure 4**].

## Discussion

Many patients with HES continue to experience disease flares despite receiving standard of care therapies such as OCS, IS, CT, or other therapies (2, 6). This *post hoc* analysis demonstrates the add-on benefit of mepolizumab on top of existing treatments as mepolizumab was associated with a lower occurrence of flares versus placebo across all baseline therapy groups. The small sample size in the No OCS/IS/CT therapy group in particular led to large confidence intervals in all analyses, although numerically greater reductions in flare occurrence were consistently seen. This provides further evidence of the benefit of mepolizumab in patients with HES and suggests that mepolizumab therapy should be considered in patients with HES irrespective of prior therapy.

The previously-reported results from the 200622 study demonstrated that the proportion of patients experiencing ≥1 flare (or withdrawing from the study) was 50% lower in patients receiving mepolizumab compared with placebo [15/54 (28%) vs 30/54 (56%)] (9). In the current analysis, results on the proportion of patients who experienced ≥1 flare over the course of the study, the rate of flare, the time to first flare, and the proportion of patients who experienced ≥1 flare during Weeks 20–32, in the IS/CT (± OCS), OCS No IS/CT, and No IS/CT/OCS baseline treatment groups all indicated a benefit of mepolizumab treatment versus placebo for flare reduction. The greatest impact of mepolizumab on flare reduction was consistently seen in patients receiving IS/CT therapy, of whom the majority were also receiving baseline OCS, and only five patients in this group did not receive OCS. Given that IS/CT therapy is usually prescribed for patients who are still symptomatic despite treatment with OCS, this group of patients may have more severe disease or be less responsive to OCS. Notably, patients receiving IS/CT (± OCS) had a shorter disease duration than patients in the other baseline therapy groups, and as such it is possible that these patients had more aggressive disease presentations than patients with a longer duration of disease in the other baseline therapy subgroups. The fact that patients remained poorly controlled by their current IS/CT and OCS therapies does not rule out the possibility of a cumulative effect of prior treatments and mepolizumab in reducing disease flares. Therefore, mepolizumab may offer particular benefit to patients with HES that is poorly controlled despite receiving a high level of medical therapy. Consistent with this hypothesis, a previous analysis of the proportion of patients experiencing a flare over the course of the study and the rate of flares by baseline OCS dosage (0, 0 to ≤5, 5> to ≤10, >10 mg/day), identified a possible trend for increased efficacy of mepolizumab in groups with higher baseline OCS dosage (9).

It is important to note that the differences in baseline HES therapy of patients in the different subgroups may be due to differences in physician treatment patterns, differences in disease severity between patients, or other considerations such as lack of efficacy or intolerance to previous treatments. In addition, as HES is a heterogeneous disease, differences in baseline therapy may reflect differences in patterns of disease course, with some patients experiencing chronic levels of inflammation requiring constant treatment, whilst others experience periodic relapses with variable time of remission between flares, and therefore may not be receiving chronic treatment (3). In the No IS/CT/OCS group, a higher portion of patients presented with abdominal pain/bloating or muscle/joint pain, while patients in the IS/CT (± OCS) and OCS No IS/CT subgroups, reported breathing and skin symptoms as their most bothersome symptoms. It should be noted that whilst 23% of patients were not receiving chronic OCS or IS/CT at baseline, all had experienced at least two flares in the 12 months prior to screening requiring episodic treatment with OCS or IS/CT. Nonetheless, overall, our results suggest that patients who did not receive any treatment may have had milder disease, and that the benefit of mepolizumab treatment may be less obvious than in patients with more severe disease.

Given the adverse effects of chronic OCS use, there is a clinical need for treatments that allow for reductions in OCS dose and improvements in disease control (12). However, the heterogeneous nature of HES complicates treatment and the individual effectiveness of OCS-sparing drugs greatly depends on the specific diagnosis (13–15). In routine clinical practice, IS/CT therapies such as azathioprine or methotrexate are sometimes used as steroid-sparing agents, particularly in idiopathic HES, with little or no evidence of effectiveness (13–15). Conversely, hydroxycarbamide, interferon-α, or PEG-interferon can be effective in patients who experience significant corticosteroid-related adverse effects or who fail to respond adequately to OCS, particularly in those with myeloid subtypes (13–15). Imatinib, an effective treatment for patients with aberrant PDGF-R signalling activation (most commonly driven by a *FIP1L1-PDGFRA* fusion gene), is sometimes given as a steroid sparing agent to patients without identified *PDGF-R* activation mutations, with highly variable treatment responses being observed (14, 16). However, the OCS-sparing effect of these therapies has not been demonstrated in randomised controlled clinical trials and their use can be limited by adverse effects (13–15). The OCS-sparing effect of mepolizumab in severe eosinophilic asthma and EGPA is well-established (17–19) and there is emerging evidence of an OCS sparing effect of mepolizumab in patients with HES (20, 21). In the 20-week open label extension of the 200622 study (205203/NCT03306043) a ≥50% reduction in OCS daily dose was achieved by 28% of patients using OCS during the first 4 weeks of the study (21). Mepolizumab may therefore offer the additional benefit of OCS reduction for patients requiring chronic or repeated courses of OCS.

The limitations of the parent 200622 study have been previously reported (9). The *post hoc* nature of the current study led to additional limitations. Owing to the rarity of HES, the numbers of patients in some of the baseline treatment subgroups were small, with 23 patients receiving IS/CT (± OCS), 60 receiving OCS No IS/CT and 25 receiving No OCS/IS/CT. Whilst the 200622 study was powered to detect an absolute reduction of 38% in the proportion of patients experiencing a flare with a sample size of 50 in each arm, this *post hoc* analysis was not powered for such testing. Statistical testing or more detailed analysis of between group differences was therefore not attempted. As such, the results of the subgroup analyses presented here should be viewed as indicative rather than definitive and further studies will be needed to fully explore the factors contributing to the patterns seen. In addition, as historical treatment data were not collected, it was not possible to investigate prior treatment use and the rationale for the treatments patients were receiving at baseline. This circumstance, together with the heterogeneous nature of HES also limits the ability to draw conclusions on any differences in disease severity between baseline therapy groups, and limits the interpretation of the results. Despite these caveats our results provide additional insights, beyond the primary results of the 200622 study (9), into the treatment benefits of mepolizumab for patients with HES receiving varying baseline therapies.

In conclusion, the results of this analysis provide further evidence of the beneficial effects of mepolizumab treatment in terms of flare reduction in patients with HES. Our findings suggest the impact of mepolizumab may be especially pronounced in patients with poor disease control despite receiving treatment with IS/CT therapies with or without OCS. Nonetheless, it is likely that patients with poorly controlled HES will benefit from treatment with mepolizumab irrespective of existing HES treatment.

## Participating investigators of the HES mepolizumab study group

Participating investigators: Gabriel Ricardo García, Adriana Sosso, Luis Wehbe, Anahí Yañez; Daniël Blockmans, Florence Roufosse, Martti Anton Antila, Daniela Blanco, Sergio Grava, Marina Andrade Lima, Andreia Luisa Francisco Pez, Stanislas Faguer, Mohamed A. Hamidou, Jean-Emmanuel Kahn, Guillaume Lefévre, Knut Brockow, Peter M. Kern, Juliana Schwabb, Bastian Walz, Tobias Welte, Fabrizio Pane, Alessandro M. Vannucchi, Ruth Cerino-Javier, Alfredo Gazca-Aguilar, Dante D. Hernández-Colín, Héctor Glenn Valdéz-López, Izabela R. Kupryś-Lipińska, Jacek Musial, Witold Prejzner, Eniko Mihaly, Viola Popov, Mihnea Tudor Zdrenghea, Sergey V. Gritsaev, Vladimir Ivanov, Nikolay Tsyba, Aránzazu Alonso, Georgina Espígol-Frigolé, Maria Laura Fox, Regina Garcia Delgado, Jesús María Hernández Rivas, Guillermo Sanz Santillana, Ana Isabel González, Andrew J. Wardlaw, Praveen Akuthota, Joseph H. Butterfield, Geoffrey L. Chupp, John B. Cox, Gerald J. Gleich, Devi Jhaveri, Marc E. Rothenberg

## Affiliation details of participating investigators in the HES mepolizumab study group

Argentina: Gabriel Ricardo García, Centro Platense en Investigaciones Respiratorias, Buenos Aires; Adriana Sosso, Centro de Investigaciones Clinicas, Buenos Aires; Luis Wehbe, Instituto Ave Pulmo-Fundacion Enfisema, Buenos Aires; Anahí Yañez, Investigaciones en Alergia Enfermedades Respiratorias-Consultorios Medicos, Buenos Aires; Belgium: Daniël Blockmans, UZ Leuven – Campus Gasthuisberg, Leuven; Florence Roufosse, Hôpital Erasme, Brussels; Brazil: Martti Anton Antila, Clinica de Alergia Martti Antila and Hospital Santa Lucinda, Sorocaba, São Paulo; Daniela Blanco, Hospital São Lucas da PUCRS, Centro de Pesquisa Clinica, Porto Alegre, Rio Grande Do Sul; Sergio Grava, Parana Medical Research Center, Maringá, Paraná; Marina Andrade Lima, Hospital Dia do Pulmão, Departamento de Pesquisa Clinica, Blumenau, Santa Catarina; Andreia Luisa Francisco Pez, Pesquisare Saúde Sociedade Simples Ltda, Santo André-SP, São Paulo; France: Stanislas Faguer, Centre Hospitalier Universitaire de Toulouse - Hôpital Rangueil, Toulouse, Mohamed A. Hamidou, Centre Hospitalier Universitaire de Nantes, Nantes; Jean-Emmanuel Kahn, Hôpital Foch, Service de Médecine Interne, Suresnes; Guillaume Lefévre, Centre Hospitalier Régional Universitaire de Lille – Hôpital Claude Huriez, Lille; Germany: Knut Brockow, Technische Universitaet Muenchen, Klinik and Poliklinik für Dermatologie, Muenchen; Peter M. Kern, Klinikum Fulda-MVZ Medizinische Klinik IV, Fulda; Juliana Schwaab, University Hospital Mannheim, Heidelberg University, Mannheim, Germany; Bastian Walz, Kreiskliniken Esslingen Klinik Kirchheim, Kirchheim unter Teck; Tobias Welte, Medizinische Hochschule, Hannover; Italy: Fabrizio Pane, Azienda Ospedaliera Universitaria Federico II, U. O. Ematologia e Trapianti di Midollo, Napoli; Alessandro M. Vannucchi, Azienda Ospedaliero-Universitaria di Careggi, Dipartimento di Medicina Sperimentale e Clinica, Firenze; Mexico: Ruth Cerino-Javier, Hospital Angeles de Villahermosa, Villahermosa, Tabasco; Dante D. Hernández-Colín, Instituto Jalisciense de Investigación Clinica, Sociedad Anónima de Capital Variable, Guadalajara, Jalisco; Héctor Glenn Valdéz-López, CRI Centro Regiomontano de Investigacion SC, Monterrey, Nuevo León; Poland: Izabela R. Kupryś-Lipińska, Samodzienly Publiczny Zaklad Opieki Zdrowotnej, Uniwersytecki Szpital Kliniczny nr 1 im. Norberta Barlickiego UM W Łodzi, Łódź, Łódźkie; Jacek Musial, Szpital Uniwersytecki w Krakowie, Oddzial Kliniczny Alergii i Immunologii, Krakow; Witold Prejzner, Uniwersyteckie Centrum Kliniczne, Klinika Hematologii i Transplantologii, Gdansk; Romania: Eniko Mihaly, SC Dora Medicals SRL Targu Mures; Viola Popov, Centrul Medical Unirea SRL – Policlinica Enescu, Bucharest; Mihnea Tudor Zdrenghea, Institutul Oncologic “Prof Dr Ion Chiricuta”, Cluj-Napoca; Russia: Sergey V. Gritsaev, Russian Hematology and Transfusiology Research Center, St Petersburg; Vladimir Ivanov, Almazov National Medical Research Center, Ministry of Health of Russian Federation, St Petersburg; Nikolay Tsyba, Scientific Advisory Department of Chemotherapy of Myeloproliferative Disorders, Hematology Research Center, Moscow; Spain: Aránzazu Alonso, Hospital Quirón Madrid Servicio de Hematologia, Madrid; Georgina Espígol-Frigolé, Hospital Clinic i Provincial de Barcelona, Barcelona; Maria Laura Fox, Hospital Vall d Hebrón, Barcelona; Regina Garcia Delgado, Hospital Virgen de la Victoria, Campus Universitario de Teatinos, Málaga; Jesús María Hernández Rivas, Hospital Universitario de Salamanca,1 °C Planta/Laborotoria de Hematología, Salamanca; Guillermo Sanz Santillana, Hospital Universitari i Politecnic La Fe, Valencia; Ana Isabel González, Hospital Universitari i Politecnic La Fe, Valencia; United Kingdom: Andrew J. Wardlaw, Institute for Lung Health, Department of Allergy and Respiratory Medicine, Glenfield Hospital, Respiratory Biomedical Research Unit, Leicester; United States: Praveen Akuthota, University of California, San Diego, California, Joseph H. Butterfield, Mayo Clinic, Rochester, Minnesota; Geoffrey L. Chupp, Yale New Haven Hospital, New Haven, Connecticut; John B. Cox, Medical University of South Carolina Investigational Drug Services, Charleston, South Carolina; Gerald J. Gleich, University of Utah, Dermatology Midvalley Health Center, Murray, Utah; Devi Jhaveri, Ohio Clinical Research Associates, Mayfield Heights, Ohio; Marc E. Rothenberg, Cincinnati Children’s Hospital Medical Center, Cincinnati, Ohio.

## Data Availability Statement

The original contributions presented in the study are included in the article/Supplementary Material. Further inquiries can be directed to the corresponding author. Anonymised individual participant data and study documents for the parent study can be requested for further research from www.clinicalstudydatarequest.com.

## Ethics Statement

For the HES 200622 study the local institutional review board or ethics committee at each study center oversaw trial conduct and documentation. The approving ethics committees were as follows: **Argentina:** Instituto Medico Platense, Boulevard 51 Nro 315, La Plata, Buenos Aires, B1900AVG, Comité de Ética en Investigación INAER, Arenales 3146 1°A, Ciudad Autonoma de Buenos Aires, Buenos Aires, C1425BEN, Comite de Etica en Investigacion, Instituto Ave Pulmo, Carlos M. Alvear 3345, Mar del Plata, Buenos Aires, B7602DCK, **Belgium:** Hôpital Erasme, Route de Lennik 808, Brussels, 1070, **Brazil:** Comite de Etica em Pesquisa do Investiga- Instituto de Pesquisas, Avenue Romeu Tortima, 739 - CEP: 13084-791, Campinas, São Paulo, 13084-791, Hospital Sao Lucas da PUCRS, Avenue Ipiranga, 6690, Porto Alegre, Rio Grande Do Sul, 90610-000, Comitê de Ética em Pesquisa - CESUMAR, Avenue Guedner, 1610 - Bloco 10 - Jardim Aclimação, Maringa, Paraná, 87050-900, CEPH - FURB, Bloco A, 2° Andar, Rua Antônio da Veiga, 140, Blumenau, Santa Catarina, 89.012-900, CEP Centro universitario Saude ABC, Avenida Principe de Gales 821, Santo Andre, São Paulo, 9060650, **France:** Comité de Protection des Personnes Ile de France III - Hôpital Tarnier, 89, rue d’Assas, Paris, 75006; **Germany:** Ethikkommission der Medizinischen Hochschule Hannover, Carl-Neuberg-Strasse 1, Hannover, Niedersachsen, 30625, **Italy:** Comitato Etico Università Federico II di Napoli, Segreteria Tecnico-Amministrativa, Edificio 20, *Via* Pansini, 5, Napoli, Campania, 80131, Comitato Etico Reg. Toscano “Area Vasta Centro”, Segreteria Scientifico-Amministrativa - Nuovo Ingresso Careggi, pad. 3 - Didattica, Largo Brambilla, 3, Firenze, Toscana, 50134, **Mexico:** Instituto Jalisciense de Investigación Clínica, Sociedad Anónima de Capital Variable, Penitenciaria 20, Guadalajara, Jalisco, 44100, Biomedical Research G And L, Sociedad De Responsabilidad Limitada De Capital Variable, Avenida La Calma 3475 Colonia La Calma, Zapopan, Jalisco, 45070, CEIIC Comite de Etica en Independiente en Investigacion Cientifica, Aguilar Sur 669 Colonia Obispado CP, Monterrey, Nuevo León, 64060, **Poland:** Komisja Bioetyczna Uniwersytetu Jagiellonskiego, Grzegorzecka 20, Krakow, 31-531, **Romania:** Comisia Nationala de Bioetica a medicamentelor si a Dispozitivelor Medicale, Pavilion K, Spitalul Clinic Colentina, Sos. Stefan cel Mare nr. 19-21, Bucuresti, 20125, **Russian Federation:** Russian Hematology and Transfusiology Research Center, 16, 2nd Sovetskaya strasse, Saint Petersburg, 191024, Almazov National Medical Research Center, 2, Akkuratova street, Saint Petersburg, 197341, Hematology Research Center, 4A, Novyi Zykovskyi proezd, Moscow, 125167, **Spain:** Hospital la Paz, Paseo de la Castellana, 261, Madrid, 28046, **UK:** East Midlands Nottingham 2, The Old Chapel, Royal Standard Place, Nottingham, NG1 6FS, **USA:** Human Research Protection Program, University of California San Diego, 9500 Gilman Drive, LaJolla, California, 92037, Mayo Institutional Review Board, Mayo Clinic, 200 First Street, South West, Rochester, Minnesota, 55905, Yale University human Investigation committee, 25 Science park – 3rd Floor, 150 Munson Street, New Haven, Connecticut, 06520, Western Institutional Review Board, 1019 39th Avenue South East, Suite 120, Puyallup, Washington, 98374, University of Utah, Institutional Review Board, Research Administration Building, 75 South 2000 East, Salt Lake City, Utah, 84112, Copernicus Group, Suite 200, 5000 Centre Green Way, Cary, North Carolina, 27513, Cincinnati Children’s Hospital Medical Center Institutional Review Board, 3333 Burnet Avenue, Location R 5392, Cincinnati, Ohio, 45229. The patients/participants provided their written informed consent to participate in this study.

## Author Contributions

EM, SY, and JS were involved in the conception or design of the analysis. AR, GL, and MC contributed to the acquisition of data. All authors were involved in the analysis or interpretation of data. All authors reviewed and revised the manuscript critically for important intellectual content, agreed to submit to the current journal, gave final approval of the version to be published, and agree to be accountable for all aspects of the work.

## Conflict of Interest

AR declares consultancy and advisory board attendance for Blueprint, Novartis, Incyte, Celgene, Abbvie and AOP, and participation as a trial investigator for Blueprint, Novartis, Incyte, Celgene, Abbvie, AOP and GSK. GL reports consulting or advisory fees from Takeda, AstraZeneca, Shire, and Sanofi Genzyme and research grant and travel and accommodation expenses from Octapharma, Takeda, and GSK, Shire. MCC has received consultancy fees from, Janssen and GSK, research grant from Kiniksa Pharmaceuticals, lecturing fees from Vifor and GSK and Roche, and Scientific meeting expenses from Roche and Kiniksa Pharmaceuticals and GSK. NK, EM, SWY and JS are all employees of GSK and own stock/shares in GSK.

This study received funding from GlaxoSmithKline. The funder had the following involvement with the study: this *post hoc* analysis, the parent study (GSK ID: 200622, Clinical Trial.gov number NCT02836496) and the editorial support provided were funded by GlaxoSmithKline.

## Publisher’s Note

All claims expressed in this article are solely those of the authors and do not necessarily represent those of their affiliated organizations, or those of the publisher, the editors and the reviewers. Any product that may be evaluated in this article, or claim that may be made by its manufacturer, is not guaranteed or endorsed by the publisher.

## Abbreviations

CT, cytotoxic; *FIP1L1-PDGFRA*, FIP1-like-1- platelet-derived growth factor receptor alpha; HES, hypereosinophilic syndrome; IS, immunosuppressive; OCS oral corticosteroids.
